# Multifactor dimensionality reduction reveals the effect of interaction between ERAP1 and IFIH1 polymorphisms in psoriasis susceptibility genes

**DOI:** 10.3389/fgene.2022.1009589

**Published:** 2022-11-08

**Authors:** Chang Zhang, Qin Qin, Yuanyuan Li, Xiaodong Zheng, Weiwei Chen, Qi Zhen, Bao Li, Wenjun Wang, Liangdan Sun

**Affiliations:** ^1^ Department of Dermatology, The First Affiliated Hospital of Anhui Medical University, Hefei, China; ^2^ Institute of Dermatology, Anhui Medical University, Hefei, China; ^3^ Key Laboratory of Dermatology, Anhui Medical University, Ministry of Education, Hefei, China; ^4^ Inflammation and Immune Mediated Diseases Laboratory of Anhui Province, Hefei, China; ^5^ Anhui Provincial Institute of Translational Medicine, Hefei, China

**Keywords:** psoriasis, gene–gene interaction, multifactor dimensionality reduction, genome-wide association studies, IFIH1, ERAP1

## Abstract

**Background:** Psoriasis is a common immune-mediated hyperproliferative skin dysfunction with known genetic predisposition. Gene–gene interaction (e.g., between HLA-C and ERAP1) in the psoriasis context has been reported in various populations. As ERAP1 has been recognized as a psoriasis susceptibility gene and plays a critical role in antigen presentation, we performed this study to identify interactions between ERAP1 and other psoriasis susceptibility gene variants.

**Methods:** We validated psoriasis susceptibility gene variants in an independent cohort of 5,414 patients with psoriasis and 5,556 controls. Multifactor dimensionality reduction (MDR) analysis was performed to identify the interaction between variants significantly associated with psoriasis in the validation cohort and ERAP1 variants. We then conducted a meta-analysis of those variants with datasets from exome sequencing, target sequencing, and validation analyses and used MDR to identify the best gene–gene interaction model, including variants that were significant in the meta-analysis and ERAP1 variants.

**Results:** We found that 19 of the replicated variants were identified with *p* < 0.05 and detected six single-nucleotide polymorphisms of psoriasis susceptibility genes in the meta-analysis. MDR analysis revealed that the best predictive model was that between the rs27044 polymorphism of ERAP1 and the rs7590692 polymorphism of IFIH1 (cross-validation consistency = 9/10, test accuracy = 0.53, odds ratio = 1.32 (95% CI, 1.09–1.59), *p* < 0.01).

**Conclusion:** Our findings suggest that the interaction between ERAP1 and IFIH1 affects the development of psoriasis. This hypothesis needs to be tested in basic biological studies.

## Introduction

Psoriasis is a common immune-mediated hyperproliferative skin dysfunction with known genetic predisposition that affects almost 125 million people worldwide (Armstrong and Read, 2020). Psoriasis morbidity rates range from 0.09% to 5.1% (Michalek et al., 2017), with major regional differences. The condition results from a combination of interacting genetic, environmental, and immunological factors (Myers et al., 2005; Mahil et al., 2015; Greb et al., 2016). Although progress has been made in the interpretation of its heredity, the pathogenesis of psoriasis remains unclear. Early correlation studies established that the genetic contribution to psoriasis is complex and multifactorial.

Since 2005, genome-wide association studies (GWASs) have been established as the most effective approach to the identification of genetic variations related to complex diseases *via* the use of a complex single-nucleotide polymorphism (SNP) map of the human genome to identify differences in allele frequency between patients and controls (Kruglyak, 2008; Zhang, 2012). Although GWASs have led to the robust identification of numerous susceptibility loci, they capture small proportions of estimated disease heritability; this issue is referred to as the “missing heritability” problem (Manolio et al., 2009; Eichler et al., 2010). Most correlational research involves the detection of SNPs in isolation, without examination of the mutual effects of their interaction, leading intricate reciprocities in the biosystem to be overlooked. With the accumulation of large amounts of genome data, further gene–gene interaction and association studies are essential to explore the biological pathways and pathogeneses of common diseases (Cordell, 2009).

Most common diseases are induced by the nonlinear interaction of numerous genetic and environmental factors. Complex diseases are always generated by gene–environment and gene–gene interactions, the latter of which can enhance their danger (Evans et al., 2006; Cordell, 2009). Many methods can be used to test for gene–gene interaction, including those involving logistic regression models, neural networks, and multifactor dimensionality reduction (MDR) (Cordell, 2009). The use of a traditional (e.g., logistic regression) model to detect multiple interactions at the same time imposes a huge computational burden due to the exponential increase in the number of interaction terms (Chattopadhyay and Lu, 2019). Although neural networks are considered good nonlinear models, they are prone to overfitting (Ritchie et al., 2003). MDR, developed by Ritchie et al. (Ritchie et al., 2001) in 2001, is a non-parametric model-free method that can be applied directly in case-control and discordant sib-pair studies. It involves no *a priori* genetic model assumption, unlike other approaches (e.g., linear regression or generalized linear models); this enables the detection of any genetic interaction in relation to disease. With the division of samples into high- and low-risk groups, MDR enables the reduction of *n*-dimensional models to one-dimension and reduces type-I and-II errors. It is currently the most popular method for detection of gene–gene interaction, and it has been used to identify potential interaction loci in many diseases, including sporadic breast cancer (Ritchie et al., 2001), hypertension (Moore and Williams, 2002), type-2 diabetes mellitus (Cho et al., 2004), and the autoimmune diseases rheumatoid arthritis (Julià et al., 2007; Liu et al., 2011), systemic lupus erythematosus (Zhang et al., 2016), and ankylosing spondylitis (Evans et al., 2011).

Based on existing GWAS data (Zhang et al., 2009), our group has analyzed the interaction of various susceptibility genes (including HLA–IL12B, HLA–ICE (Zheng et al., 2011), HLA-C–ERAP1, and HLA-C–TRAF3IP2 (Yin et al., 2013) interactions) in the Han Chinese population. Such gene interactions, including those related to psoriasis, have been reported in different populations (Strange et al., 2010). ERAP1 has been reported as being related to psoriasis in multiple populations, and our group has genotyped and replicated its polymorphisms (Chen et al., 2022). In this study, we detected interactions of ERAP1 with other psoriasis susceptibility genes in the Han Chinese population.

## Materials and methods

### Sample collection

This study was conducted with data generated in our previous research—an exome sequencing study conducted with 781 patients with psoriasis and 676 controls and a targeted sequencing study conducted with 9,946 psoriasis cases and 9,906 controls (Tang et al., 2014). We performed a case-control study with an independent sample cohort consisting of 5,414 psoriasis cases and 5,556 controls to validate the psoriasis susceptibility genes. All patients and controls were of Han Chinese origin and had attended the First Affiliated Hospital of Anhui Medical University. The controls were healthy volunteers without psoriasis, other autoimmune diseases, systemic disease, or a family history of psoriasis (in first-, second-, and third-degree relatives). Each patient was examined by medical professionals and diagnosed with psoriasis by two experienced dermatologists, with rigorous recording of clinical data to ensure their specificity and reliability. After obtaining informed consent, medical professionals collected peripheral blood samples from all patients and controls. This research was approved by the Ethics Committee of Anhui Medical University Committee and performed following Declaration of Helsinki guidelines.

### Procedure

Genomic DNA was extracted from the participants’ peripheral blood mononuclear cells. DNA concentrations and optical density ratios (A_260_/A_280_) were determined using a NanoDrop 1,000 spectrophotometer (Thermo Fisher Scientific, United States). The Sequenom MassArray system (Sequenom, United States) was used for SNP genotyping. We used 15 ng standardized genomic DNA per sample for subsequent genetic typing. We used multiplex polymerase chain reactions to amplify the genomic DNA and then performed site-specific monacyl elongation reactions to obtain products. The amplified products were purified with AgencourtAM SPRI XP microbeads (Beckman Coulter Life Sciences, United States). After desalination, the final products were transferred to a 384-element SpectrCHIP array (Applied Biosystems, United States). Alleles were detected with a matrix-assisted laser desorption ionization time-of-flight 70 mass spectrometer and analyzed using Massarray Typer 71 software (Sequenom, United States). In the independent cohort, validated SNPs were selected using the following terms: position within 500 kb of a psoriasis susceptibility gene tag, *p* < 1.0 × 10^−4^ in the exome sequencing and targeted sequencing studies, genotyping detection rate >90%, and Hardy–Weinberg test *p* > 1.0 × 10^−4^ in controls.

### Gene–gene interaction analysis

Based on our group’s previous study (Chen et al., 2022), ERAP1 variants were genotyped and replicated in the present cohort. The statistical analysis was performed with PLINK 1.07 and a significance level of *p* < 0.05. Gene–gene interactions were analyzed with open-source MDR software (ver. 2.0 Beta 2; available from http://sourceforge.net/projects/mdr). As the MDR software requires complete datasets with no missing value, we replaced missing values with numbers that did not represent genotypes; the second method used was *k*-nearest neighbor estimation to fill in the missing values (*k* was equal to the square root of the number of SNPs) (Schwender, 2012).

### Meta-analysis of GWAS discovery results

To expand the sample size and detect more gene–gene interactions, we conducted a meta-analysis of summary GWAS statistics from the exome sequencing (*n* = 1,457), targeted sequencing (*n* = 19,007), and validation (*n* = 10,970) cohorts. We performed the analysis with METAL (available from http://www.sph.umich.edu/csg/abecasis/Metal/), with the test statistics weighted by sample size. The genome-wide significance threshold was *p* < 5 × 10^−8^. We performed genomic correction of all results to control for potential inflation of the test statistics. Effector allele frequencies were tracked selectively across all files.

## Results

### Independent cohort validation

Of 39 SNPs selected for genotyping verification, 19 were significant (*p* < 0.05) in the independent cohort validation analysis. IFIH1 showed the strongest correlation with psoriasis (chr_163737871, *p* = 6.52 × 10^−7^, odds ratio (OR) = 0.81; chr2_163136771, *p* = 1.72 × 10^−6^, OR = 0.81; Table 1). Other validated psoriasis susceptibility genes were ERAP2 (5q15), IL18R1 (2q12.1), LTB (12p13.31), IL1RL1 (2q12.1), CARD14 (17q25.3), and SLC9A4 (2q12.1).

**TABLE 1:** SNPs with significance in replication analysis.

Chr.	Variant ID	Gene	F_A	F_U	Allele	*p* value	OR (95% CI)
2q24.3	rs12479043	IFIH1	0.10	0.12	G/C	6.52 × 10^−7^	0.81 (0.74–0.88)
2q24.3	rs7590692	IFIH1	0.10	0.12	C/T	1.72 × 10^−6^	0.81 (0.74–0.88)
5q15	rs2303208	ERAP2	0.37	0.40	A/G	9.75 × 10^−6^	0.88 (0.83–0.93)
2q12.1	rs1882348	IL18R1	0.32	0.35	A/T	2.56 × 10^−5^	0.89 (0.84–0.94)
12p13.31	rs12354	LTBR	0.12	0.14	T/G	3.85 × 10^−5^	0.85 (0.78–0.92)
2q12.1	rs873022	IL1RL1	0.29	0.32	T/G	4.73 × 10^−5^	0.88 (0.83–0.94)
17q25.3	rs4889997	CARD14	0.49	0.51	A/G	5.03 × 10^−5^	0.89 (0.85–0.94)
2q12.1	rs61731285	SLC9A4	0.11	0.09	T/C	8.55 × 10^−5^	1.20 (1.09–1.31)
2q12.1	rs3213733	IL18R1	0.11	0.10	A/C	9.29 × 10^−5^	1.19 (1.09–1.30)
2q12.1	rs12905	IL1RL1	0.30	0.32	A/G	1.03 × 10^−4^	0.89 (0.84–0.94)
2q12.1	rs3213732	IL18R1	0.17	0.15	G/A	8.66 × 10^−4^	1.14 (1.05–1.23)
2q12.1	rs2287033	IL18R1	0.17	0.15	C/T	1.23 × 10^−3^	1.13 (1.05–1.22)
2q12.1	rs6749014	IL18R1	0.16	0.15	T/C	2.18 × 10^−3^	1.12 (1.04–1.21)
2q12.1	rs4988956	IL1RL1	0.15	0.13	A/G	5.08 × 10^−3^	1.12 (1.03–1.21)
4q24	rs3817685	NFKB1	0.40	0.38	G/C	5.34 × 10^−3^	1.08 (1.02–1.14)
1q32.1	rs28694304	C1orf186	0.13	0.12	A/C	8.51 × 10^−3^	1.12 (1.03–1.21)
13q12.11	rs72474224	GJB2	0.05	0.05	T/C	2.19 × 10^−2^	1.15 (1.02–1.30)
19p13.2	rs280497	TYK2	0.42	0.41	A/G	2.78 × 10^−2^	1.06 (1.01–1.12)
17q25.3	rs2066964	CARD14	0.46	0.47	C/G	3.68 × 10^−2^	0.94 (0.89–1.00)

Chr. chromosome, F_A frequency in cases, F_U frequency in controls.

### Gene–gene interaction study

**FIGURE 1:**
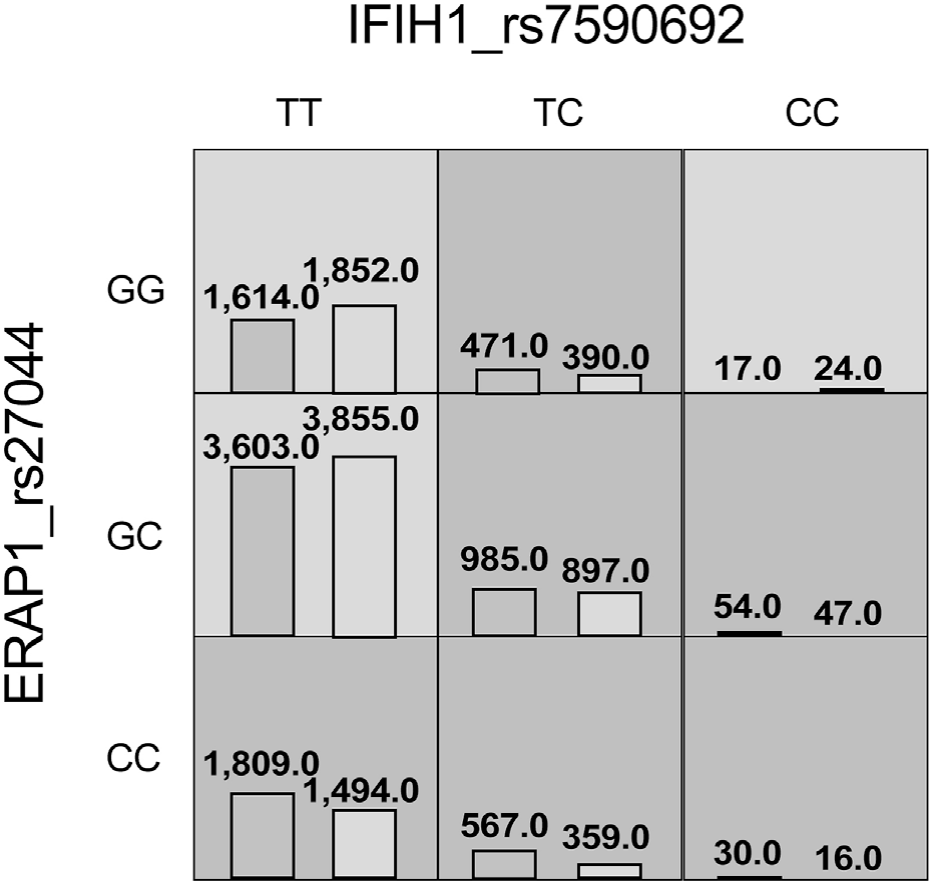
High- and low-risk genotypes in the best psoriasis interaction model. Dark-gray cells represent high risk (combinations exceeding the *r* ratio of the percentage of cases to controls ≥1.0) and light-gray cells represent low risk (combinations below the ratio threshold).

**FIGURE 2:**
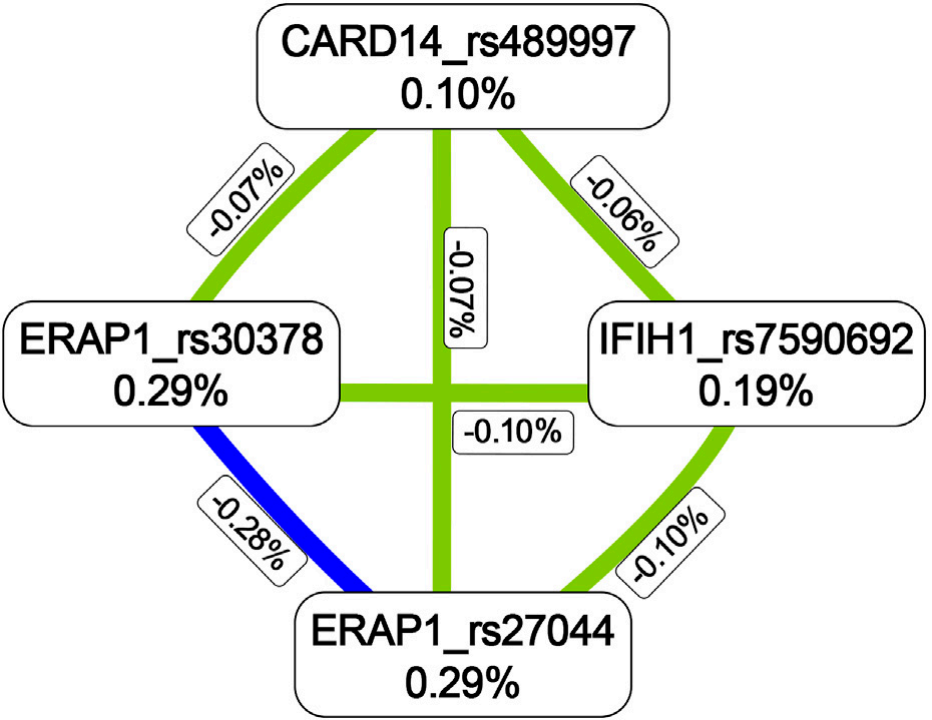
Circular graph of the best models. The numbers between two SNPs was information gain, which was used to detect and characterize epistatics of the interaction model and calculated by constructing two attributes using the MDR function. Red: positive information gain (synergistic or non-additive effect); blue: negative information gain (redundancy or correlation); yellow: no information gain (independence).

**FIGURE 3:**
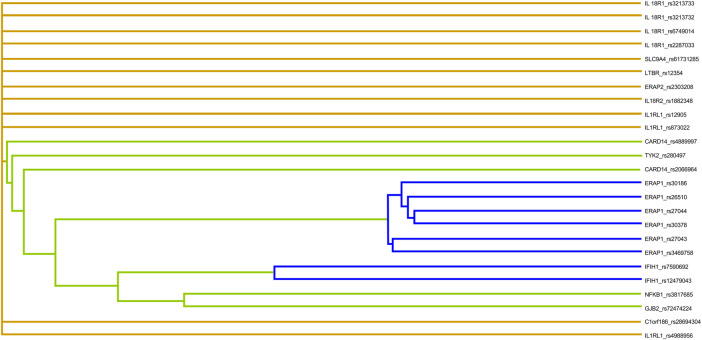
Dendrogram between polymorphisms in psoriasis susceptibility genes. Colors depict degrees of synergy, ranging from red (most information gain) to yellow–green and blue (most information redundancy).

The MDR analysis was conducted with the 19 SNPs showing significance in the replicated cohort and six ERAP1 SNPs. The rs27044 polymorphism of ERAP1 was the best single-locus model (CVC = 10, test accuracy = 0.52, OR = 1.32 (95% CI, 1.07–1.63), *p* < 0.01). The best predictive model (i.e., that with maximum testing accuracy) was that between the rs27044 polymorphism of ERAP1 and the rs7590692 polymorphism of IFIH1 (CVC = 9/10, test accuracy = 0.53, OR = 1.32 (95% CI, 1.09–1.59), *p* < 0.01; Table 2). In this model, high-risk genotypes were TT × CC, TC × GG, TC × GC, TC × CC, CC × GC, and CC × CC, and low-risk genotypes were TT × GC, TT × GG, and CC × GG (Figure 1). Figures 2, 3 show measures of characterized epistatic of the models from the interaction analysis.

**TABLE 2:** Best multifactor dimensionality reduction (MDR) interaction models.

Locus number	Number of the risk factors (best interaction model)	Testing accuracy	CVC	0R	95% CI	*p*
1	ERAP1_rs27044	0.5231	10/10	1.32	1.06–1.64	0.0098
2	IFIH1_rs7590692,ERAP1_rs27044	0.5314	9/10	1.32	1.09–1.59	0.0036
3	IFIH1_rs7590692,ERAP1_rs27044,CARD14_rs4889997	0.5265	6/10	1.25	1.04–1.49	0.0147

The model with the maximum testing accuracy was considered the best model. MDR, multifactor dimensionality reduction; CVC, cross-validation consistency.

### Results of the meta-analysis and MDR

In the meta-analysis, six of the 39 SNPs reached the genome-wide significance threshold (*p* < 10^−8^). These SNPs were in IFIH1 (rs12479043, *p* = 2.85 × 10^−15^; rs7590692, *p* = 1.57 × 10^−14^), CARD14 (rs4889997, *p* = 4.03 × 10^−12^), GJB2 (rs72474224, *p* = 8.22 × 10^−11^), NFKB1 (rs3817685, *p* = 4.24 × 10^−10^), and ERAP2 (rs2303208, *p* = 7.50 × 10^−10^; Table 3). In the MDR analysis of these SNPs and six SNPs of ERAP1, the best model was still that between the rs27044 polymorphism of ERAP1 and the rs7590692 polymorphism of IFIH1 (CVC = 9/10, test accuracy = 0.53, OR = 1.32 (95% CI, 1.09–1.59) *p* < 0.01).

**TABLE 3:** Result of meta-analysis.

Variant ID	Gene	Chr.	A1	A2	Freq1	P_Meta_	Direction	I^2^	P_Het_
rs12479043	IFIH1	2q24.3	C	G	0.88	2.85 × 10^−15^	+++	0	0.91
rs7590692	IFIH1	2q24.3	T	C	0.88	1.57 × 10^−14^	-++	7.1	0.34
rs4889997	CARD14	17q25.3	A	G	0.50	4.03 × 10^−12^	---	65.8	0.05
rs72474224	GJB2	13q12.11	T	C	0.05	8.22 × 10^−11^	+++	59.9	0.08
rs3817685	NFKB1	4q24	C	G	0.63	4.24 × 10^−10^	?--	34.5	0.22
rs2303208	ERAP2	5q15	A	G	0.40	7.50 × 10^−10^	+--	21.5	0.28

Chr.: chromosome; A1: minor allele; A2: major allele; Freq1: frequency of allele 1; P_Meta_: *p* value of the meta-analysis; P_Het_: *p* value of heterogeneity.

## Discussion

With the deep development of psoriasis-related genetic analysis, our group has generated systemic genomic data on psoriasis through exome and targeted sequencing. In this study, we verified the psoriasis susceptibility genes IFIH1 (2q24.3), ERAP2 (5q15), IL18R1 (2q12.1), LTBR (12p13.31), IL1RL1 (2q12.1), CARD14 (17q25.3), and SLC9A4 (2q12.1). We found that the most significant interaction was between the rs27044 polymorphism of ERAP1 and the rs7590692 of IFIH1; this result was confirmed in the MDR analysis.

ERAP1 is an immune-related gene encoding multifunctional aminopeptidase that is induced by interferon-γ. It plays critical roles in antigen presentation and N-terminal peptide pruning to reach the best presenting size of the major histocompatibility complex I molecule. These molecules can present polypeptides containing 8–10 amino acids directly outside of cells, enhancing antigen presentation and T-cell activation in disease processes (Kochan et al., 2011). ERAP1 is more likely to be present in individuals carrying the HLA-C susceptibility allele, with the first and most important psoriasis susceptibility gene identified (Bowcock, 2005). A recent study revealed that ERAP1 causes psoriasis by affecting HLA-C production *via* melanocyte autoantigens (Arakawa et al., 2021). Thus, it may cause psoriasis lesions by interacting with HLA-C. ERAP1 has been related to psoriasis in Chinese (rs151823) (Sun et al., 2010) and European (rs27524) (Strange et al., 2010) populations. In a meta-analysis of nine case-control studies, the rs27044 polymorphism of ERAP1 was associated significantly with psoriasis (Wu and Zhao, 2021). This gene has also been associated with ankylosing spondylitis (Tsui et al., 2010), Behcet’s disease, multiple sclerosis, and inflammatory bowel disease (Reeves and James, 2018).

We replicated IFIH1 in our independent sample cohort with *p* = 6.52 × 10^−7^. IFIH1 encodes MDA5, an innate pattern recognition receptor. MDA5 is a cytoplasmic sensor of picornavirus nucleic acids that triggers a cascade of antiviral responses, including the induction of type I interferons and pro-inflammatory cytokines. Once it is bound to pathogen-like viral RNA, activated MDA5 interacts with mitochondrial antiviral-signaling proteins and then phosphorylated interferon regulatory factors (IRFs) 3 and 7. Dimers are formed by phosphorylated IRF-3 and -7 and move into cell nuclei to stimulate the secretion of type 1 interferon (IFN1) (Brisse and Ly, 2019). IFN1 mediates autoimmune responses to invaders such as viruses. MDA5 has been observed to significantly increase psoriatic plaques and keratinocytes, and its overexpression acts as a negative regulator for the differentiation of calcium-induced keratinocytes in psoriatic skin lesions (Hong et al., 2021). IFIH1 has been related to psoriasis in a European population (rs17716942) (Strange et al., 2010), and its rs35667974 and rs10930046 polymorphisms were found to have protective effects against psoriasis in a white North American population (Li et al., 2010). In addition, evidence supports the involvement of IFIH1 SNPs in inflammatory diseases such as type 1 diabetes (Nejentsev et al., 2009), inflammatory bowel disease (Cananzi et al., 2021), and dermatomyositis (Kurtzman and Vleugels, 2018).

Our MDR analysis revealed a significant association between ERAP1 and IFIH1, suggesting that the presence of the ERAP1 rs27044 polymorphism affects the expression of the IFIH1 rs7590692 polymorphism. In an *in vitro* experiment (Aldhamen et al., 2013), increased levels of proinflammatory cytokines such as tumor necrosis factor (TNF)-α were observed in ERAP1-knockout mice, suggesting that the lack of ERAP1 expression induces increased autoimmune activity. TNF-α, a cytokine commonly elevated in patients with psoriasis, promotes the transcription of MDA5 and RIG-I in keratinocytes (Kitamura et al., 2007; Racz et al., 2011). Taken together, these molecular biological findings suggest that ERAP1 might regulate the expression of IFIH1, but more basic biological studies are needed to determine how this gene interaction affects psoriasis.

This study has several limitations. The results provide evidence of correlations between gene–gene interactions and psoriasis but no information about the mechanisms of resulting effects, and the accuracy of the best model was not high. We will carry out further basic biological studies to verify this result. In addition, the study was conducted with data from a Han Chinese population; similar studies need to be conducted with data from other ethnic groups.

In summary, we screened for interactions between genes that affect psoriasis susceptibility and found that the most significant interaction was between the rs27044 polymorphism of ERAP1 and the rs7590692 polymorphism of IFIH1. Psoriasis is a multifactorial autoimmune disease with many genetic contributions. Although our comprehension of the genetic mechanisms underlying it has increased and the interaction of susceptibility genes has been identified based on SNPs, many questions remain. A better understanding of how these gene interactions affect the development of psoriasis, generated from basic biological studies, may provide answers to these questions.

## Data Availability

The datasets presented in this study can be found in online repositories. The names of there pository/repositories and accession number(s) can be found below: Zenodo with accession 7260050—(https://doi.org/10.5281/zenodo.7260050).

## Abbreviations

GWAS, genome-wide association studies; KNN, K-nearest neighbors; MAF, minor allele frequency; MDR, multifactor dimensionality reduction; PCR, polymerase chain reaction; SNP, single-nucleotide polymorphism; SNVs, single-nucleotide variations.

## References

[B1] Aldhamen Y. A. Seregin S. S. Rastall D. P. W. Aylsworth C. F. Pepelyayeva Y. Busuito C. J., (2013). Endoplasmic reticulum aminopeptidase-1 functions regulate key aspects of the innate immune response. PloS one 8 (7), e69539. 10.1371/journal.pone.0069539 23894499 PMC3722114

[B2] Arakawa A. Reeves E. Vollmer S. Arakawa Y. He M. Galinski A. (2021). ERAP1 controls the autoimmune response against melanocytes in psoriasis by generating the melanocyte autoantigen and regulating its amount for HLA-C*06:02 presentation. J. Immunol. 207 (9), 2235–2244. 10.4049/jimmunol.2100686 34580106 PMC7611875

[B3] Armstrong A. W. Read C. (2020). Pathophysiology, clinical presentation, and treatment of psoriasis: A review. JAMA 323 (19), 1945–1960. 10.1001/jama.2020.4006 32427307

[B4] Bowcock A. M. (2005). The genetics of psoriasis and autoimmunity. Annu. Rev. Genomics Hum. Genet. 6, 93–122. 10.1146/annurev.genom.6.080604.162324 16124855

[B5] Brisse M. Ly H. (2019). Comparative structure and function analysis of the RIG-I-like receptors: RIG-I and MDA5. Front. Immunol. 10, 1586. 10.3389/fimmu.2019.01586 31379819 PMC6652118

[B6] Cananzi M. Wohler E. Marzollo A. Colavito D. You J. Jing H., (2021). IFIH1 loss-of-function variants contribute to very early-onset inflammatory bowel disease. Hum. Genet. 140 (9), 1299–1312. 10.1007/s00439-021-02300-4 34185153 PMC8423350

[B7] Chattopadhyay A. Lu T. P. (2019). Gene-gene interaction: The curse of dimensionality. Ann. Transl. Med. 7 (24), 813. 10.21037/atm.2019.12.87 32042829 PMC6989881

[B8] Chen W. Yong L. Ge H. Xu Q. Zhen Q. Li B., (2022). Polymorphisms in ERAP1 gene are associated with psoriasis. Meta Gene 31, 100995. 10.1016/j.mgene.2021.100995

[B9] Cho Y. M. Ritchie M. D. Moore J. H. Park J. Y. Lee K. U. Shin H. D., (2004). Multifactor-dimensionality reduction shows a two-locus interaction associated with Type 2 diabetes mellitus. Diabetologia 47 (3), 549–554. 10.1007/s00125-003-1321-3 14730379

[B10] Cordell H. J. (2009). Detecting gene-gene interactions that underlie human diseases. Nat. Rev. Genet. 10 (6), 392–404. 10.1038/nrg2579 19434077 PMC2872761

[B11] Eichler E. E. Flint J. Gibson G. Kong A. Leal S. M. Moore J. H., (2010). Missing heritability and strategies for finding the underlying causes of complex disease. Nat. Rev. Genet. 11 (6), 446–450. 10.1038/nrg2809 20479774 PMC2942068

[B12] Evans D. M. Marchini J. Morris A. P. Cardon L. R. (2006). Two-stage two-locus models in genome-wide association. PLoS Genet. 2 (9), e157. 10.1371/journal.pgen.0020157 17002500 PMC1570380

[B13] Evans D. M. Spencer C. C. A. Pointon J. J. Su Z. Harvey D. Kochan G., (2011). Interaction between ERAP1 and HLA-B27 in ankylosing spondylitis implicates peptide handling in the mechanism for HLA-B27 in disease susceptibility. Nat. Genet. 43 (8), 761–767. 10.1038/ng.873 21743469 PMC3640413

[B14] Greb J. E. Goldminz A. M. Elder J. T. Lebwohl M. G. Gladman D. D. Wu J. J., (2016). Nat. Rev. Dis. Prim. 2, 16082. 10.1038/nrdp.2016.82 27883001

[B15] Hong D. K. Choi M. R. Hwang Y. L. Lee J. K. Lee Y. Seo Y. J., (2021). Potential role of cytosolic RNA sensor MDA5 as an inhibitor for keratinocyte differentiation in the pathogenesis of psoriasis. Ann. Dermatol. 33 (4), 339–344. 10.5021/ad.2021.33.4.339 34341635 PMC8273324

[B16] Julià A. Moore J. Miquel L. Alegre C. Barcelo P. Ritchie M., (2007). Identification of a two-loci epistatic interaction associated with susceptibility to rheumatoid arthritis through reverse engineering and multifactor dimensionality reduction. Genomics 90 (1), 6–13. 10.1016/j.ygeno.2007.03.011 17482423

[B17] Kitamura H. Matsuzaki Y. Kimura K. Nakano H. Imaizumi T. Satoh K., (2007). Cytokine modulation of retinoic acid-inducible gene-I (RIG-I) expression in human epidermal keratinocytes. J. Dermatol. Sci. 45 (2), 127–134. 10.1016/j.jdermsci.2006.11.003 17182220

[B18] Kochan G. Krojer T. Harvey D. Fischer R. Chen L. Vollmar M., (2011). Crystal structures of the endoplasmic reticulum aminopeptidase-1 (ERAP1) reveal the molecular basis for N-terminal peptide trimming. Proc. Natl. Acad. Sci. U. S. A. 108 (19), 7745–7750. 10.1073/pnas.1101262108 21508329 PMC3093473

[B19] Kruglyak L. (2008). The road to genome-wide association studies. Nat. Rev. Genet. 9 (4), 314–318. 10.1038/nrg2316 18283274

[B20] Kurtzman D. J. B. Vleugels R. A. (2018). Anti-melanoma differentiation-associated gene 5 (MDA5) dermatomyositis: A concise review with an emphasis on distinctive clinical features. J. Am. Acad. Dermatol. 78 (4), 776–785. 10.1016/j.jaad.2017.12.010 29229575

[B21] Li Y. Liao W. Cargill M. Chang M. Matsunami N. Feng B. J., (2010). Carriers of rare missense variants in IFIH1 are protected from psoriasis. J. Invest. Dermatol. 130 (12), 2768–2772. 10.1038/jid.2010.214 20668468 PMC3680368

[B22] Liu C. Ackerman H. H. Carulli J. P. (2011). A genome-wide screen of gene-gene interactions for rheumatoid arthritis susceptibility. Hum. Genet. 129 (5), 473–485. 10.1007/s00439-010-0943-z 21210282

[B23] Mahil S. K. Capon F. Barker J. N. (2015). Genetics of psoriasis. Dermatol. Clin. 33 (1), 1–11. 10.1016/j.det.2014.09.001 25412779

[B24] Manolio T. A. Collins F. S. Cox N. J. Goldstein D. B. Hindorff L. A. Hunter D. J., (2009). Finding the missing heritability of complex diseases. Nature 461 (7265), 747–753. 10.1038/nature08494 19812666 PMC2831613

[B25] Michalek I. M. Loring B. John S. M. (2017). A systematic review of worldwide epidemiology of psoriasis. J. Eur. Acad. Dermatol. Venereol. 31 (2), 205–212. 10.1111/jdv.13854 27573025

[B26] Moore J. H. Williams S. M. (2002). New strategies for identifying gene-gene interactions in hypertension. Ann. Med. 34 (2), 88–95. 10.1080/07853890252953473 12108579

[B27] Myers A. Kay L. J. Lynch S. A. Walker D. J. (2005). Recurrence risk for psoriasis and psoriatic arthritis within sibships. Rheumatol. Oxf. 44(6): p. 773–776. 10.1093/rheumatology/keh589 15757963

[B28] Nejentsev S. Walker N. Riches D. Egholm M. Todd J. A. (2009). Rare variants of IFIH1, a gene implicated in antiviral responses, protect against type 1 diabetes. Science 324 (5925), 387–389. 10.1126/science.1167728 19264985 PMC2707798

[B29] Racz E. Prens E. P. KantM., FlorEncia E. Jaspers N. G. Laman J. D., (2011). Narrowband ultraviolet B inhibits innate cytosolic double-stranded RNA receptors in psoriatic skin and keratinocytes. Br. J. Dermatol. 164 (4), 838–847. 10.1111/j.1365-2133.2010.10169.x 21143460

[B30] Reeves E. James E. (2018). The role of polymorphic ERAP1 in autoinflammatory disease. Biosci. Rep. 38 (4), BSR20171503. 10.1042/BSR20171503 30054427 PMC6131210

[B31] Ritchie M. D. Hahn L. W. RoodiN., Bailey L. R. Dupont W. D. Parl F. F. (2001). Multifactor-dimensionality reduction reveals high-order interactions among estrogen-metabolism genes in sporadic breast cancer. Am. J. Hum. Genet. 69 (1), 138–147. 10.1086/321276 11404819 PMC1226028

[B32] Ritchie M. D. White B. C. Parker J. S. Hahn L. W. Moore J. H. (2003). Optimization of neural network architecture using genetic programming improves detection and modeling of gene-gene interactions in studies of human diseases. BMC Bioinforma. 4, 28. 10.1186/1471-2105-4-28 PMC183838 12846935

[B33] Schwender H. (2012). Imputing missing genotypes with weighted k nearest neighbors. J. Toxicol. Environ. Health. A 75 (8-10), 438–446. 10.1080/15287394.2012.674910 22686303

[B34] Strange A. Capon F. Spencer C. C. A. Knight J. Weale M. E. Allen M. H. (2010). A genome-wide association study identifies new psoriasis susceptibility loci and an interaction between HLA-C and ERAP1. Nat. Genet. 42 (11), 985–990. 10.1038/ng.694 20953190 PMC3749730

[B35] Sun L.-D. Cheng H. Wang Z. X. Zhang A. P. Wang P. G. Xu J. H., (2010). Association analyses identify six new psoriasis susceptibility loci in the Chinese population. Nat. Genet. 42 (11), 1005–1009. 10.1038/ng.690 20953187 PMC3140436

[B36] Tang H. Jin X. Li Y. Jiang H. Tang X. Yang X., (2014). A large-scale screen for coding variants predisposing to psoriasis. Nat. Genet. 46 (1), 45–50. 10.1038/ng.2827 24212883

[B37] Tsui F. W. L. Haroon N. Reveille J. D. Rahman P. Chiu B. Tsui H. W., (2010). Association of an ERAP1 ERAP2 haplotype with familial ankylosing spondylitis. Ann. Rheum. Dis. 69 (4), 733–736. 10.1136/ard.2008.103804 19433412

[B38] Wu X. Zhao Z. (2021). Associations between ERAP1 gene polymorphisms and psoriasis susceptibility: A meta-analysis of case-control studies. Biomed. Res. Int. 2021, 5515868. 10.1155/2021/5515868 34395615 PMC8355978

[B39] Yin X. Y. Zhang R. Cheng H. Pan Q. Shen C. B. Fan X., (2013). Gene-gene interactions between HLA-C, ERAP1, TNFAIP3 and TRAF3IP2 and the risk of psoriasis in the Chinese Han population. Br. J. Dermatol. 169 (4), 941–943. 10.1111/bjd.12442 23701417

[B40] Zhang X. (2012). Genome-wide association study of skin complex diseases. J. Dermatol. Sci. 66 (2), 89–97. 10.1016/j.jdermsci.2012.02.017 22480995

[B41] Zhang X. J. Huang W. Yang S. Sun L. D. Zhang F. Y. Zhu Q. X., (2009). Psoriasis genome-wide association study identifies susceptibility variants within LCE gene cluster at 1q21. Nat. Genet. 41 (2), 205–210. 10.1038/ng.310 19169255

[B42] Zhang Y. Yang J. Zhang J. Sun L. Hirankarn N. Pan H. F., (2016). Genome-wide search followed by replication reveals genetic interaction of CD80 and ALOX5AP associated with systemic lupus erythematosus in Asian populations. Ann. Rheum. Dis. 75 (5), 891–898. 10.1136/annrheumdis-2014-206367 25862617

[B43] Zheng H. F. Zuo X. B. Lu W. S. Li Y. Cheng H. Zhu K. J., (2011). Variants in MHC, LCE and IL12B have epistatic effects on psoriasis risk in Chinese population. J. Dermatol. Sci. 61 (2), 124–128. 10.1016/j.jdermsci.2010.12.001 21208785

